# Toward a greener approach to detect inorganic arsenic using the Gutzeit method and X‐ray fluorescence spectroscopy

**DOI:** 10.1002/ansa.202200014

**Published:** 2022-09-16

**Authors:** Helen Lin, Haochen Dai, Lili He

**Affiliations:** ^1^ Department of Food Science University of Massachusetts Amherst Massachusetts 01003 USA

**Keywords:** gutzeit method, inorganic arsenic, silver nitrate, X‐ray fluorescence spectroscopy

## Abstract

Inorganic arsenic is a carcinogen repeatedly found in water and foods threatening global human health. Prior work applied the Gutzeit method and X‐ray fluorescence spectroscopy to quantify inorganic arsenic based on a harmful chemical, i.e., mercury bromide, to capture the arsine gas. In this project, we explored silver nitrate as an alternative to mercury bromide for the capture and detection of inorganic arsenic. To compare the performance of mercury bromide and silver nitrate, two standard curves were established in the range from 0 to 33.3 µg/L after optimization of reaction conditions such as the quantity of reagents and reaction time. Our result shows silver nitrate‐based standard curve had a lower limit of detection and limit of quantification at 1.02 µg/L and 3.40 µg/L, respectively, as compared to the one built upon mercury bromide that has limit of detection of 4.86 µg/L and limit of quantification of 16.2 µg/L. The relative higher sensitivity when using silver nitrate was contributed by the less interfering elements for X‐ray fluorescence analysis and thus lower background signals. A commercial apple juice was studied for matrix inference, and the results show 85%–99% recoveries and 7.4%–24.5% relative standard deviation. In conclusion, we demonstrated silver nitrate is a better choice in terms of safety restrictions and detection capability at lower inorganic arsenic concentrations.

## 1 INTRODUCTION

Arsenic is a heavy metal element with a long‐standing history of use as a poison. It is known to negatively affect neurodevelopment and cause metabolic diseases when exposed in early childhood.^1^, ^2^ Its mutagenic properties induce tumors in prostate, bladder, lung, and skin.^3^, ^4^, ^5^ Pollution sources are from both anthropogenic and geogenic activities. Volcanic eruptions, mining, smelting, and leachate bring out arsenic from the Earth's crust.^6^, ^7^, ^8^ Rocks containing arsenic dissolve in underground water. Countries such as Bangladesh, India, China, Chile, and South Africa specifically are severely affected by naturally contaminated groundwater.^9^, ^10^ The US Environmental Protection Agency's set standard (US EPA) and World Health Organization's (WHO) recommendation for maximum allowable arsenic contamination level in drinking water at 10 µg/L.^11^, ^12^

A commonly used method for quantifying iAs in lab settings is the use of high‐performance liquid chromatography (HPLC) for iAs speciation and quantified by inductively coupled plasma mass spectroscopy (ICP‐MS) as it is the most sensitive and accurate.^13^ Some disadvantages of HPLC‐ICP‐MS are expensive cost and maintenance and the requirement of trained personnel for usage. It is also inefficient for rapid in situ detection as the instrument requires a laboratory setting. For rapid in situ detection purposes, commercial kits use the Gutzeit method and colorimetric method for quantification.^14^, ^15^ This method is not always accurate for complex matrices due to color interferences by hydrogen sulfide, resulting in false positives.

X‐ray fluorescence spectrometer is a non‐destructive, rapid, and elemental‐based detection and quantification instrument.^16^ Sample atom absorbs photon from the instrument and emits an electron from the inner shell, known as the photoelectric effect. An electron from the outer shell fills the vacancy and releases energy as a fluorescent X‐ray that is unique to each atom. The energy is then measured and analyzed for identification and quantification.^17^ The instrument can distinguish and quantify multiple elements simultaneously. The peak energy on the spectra tells the element identity while the height indicates concentration. There is portable XRF which makes in situ iAs quantification possible.^18^ Green chemistry, also known as sustainable chemistry, reduces the production and use of hazardous substances to prevent waste, use safer reagents and reaction conditions, and less environmental impact after use. The challenge exists in lower reaction efficiency due to mild reaction conditions and reagents.

Prior work has established a method to quantify iAs using the Gutzeit method where arsine gas is captured by commercial mercury bromide test strips.^19^ The test strips were analyzed using XRF and quantification was achieved through establishment of a standard curve. Mercury bromide has acute oral, dermal, and inhalation toxicity.^9^ It is ranked top ten chemicals with serious health concerns by WHO.^20^ Inorganic mercury causes renal, neurological, and psychological damages.^21^ Dermal contact induces rash and contact dermatitis. For safety purposes, mercury bromide test strips were purchased instead of laboratory made in this study.

The objective of this study is to explore the potential of replacing mercury bromide with silver nitrate, which is much less toxic, to the operating personnel, thus more suitable in the further development of a field‐deployable method for iAs. Another reason for choosing silver nitrate in this study is the observation in the prior study regarding the partial overlap between mercury and arsenic. We hypothesized the use of silver nitrate could reduce the background interference. The limit of detection and linearity of standard curves obtained by both chemicals were compared. The recoveries and relative standard deviations of the silver nitrate method were investigated using apple juice as a model matrix.

## 2 MATERIALS AND METHODS

### 2.1 Materials

Zinc powder (<150 µm, 99.995% trace metal basis), tin (II) chloride (SnCl_2_, reagent grade, 98%), silver nitrate (ACS reagent, >99.0%), and mercury bromide strips were purchased from Sigma Aldrich (St. Louis, USA). Arsenic trioxide (As^3+^, 1000 µg/mL) was purchased from SPEX CertiPrep), and sulfuric acid was purchased from Certified ACS Plus. Gerber apple juice was purchased from grocery stores.

### 2.2 iAs extraction by the Gutzeit method combined with XRF measurement

**FIGURE 1:**
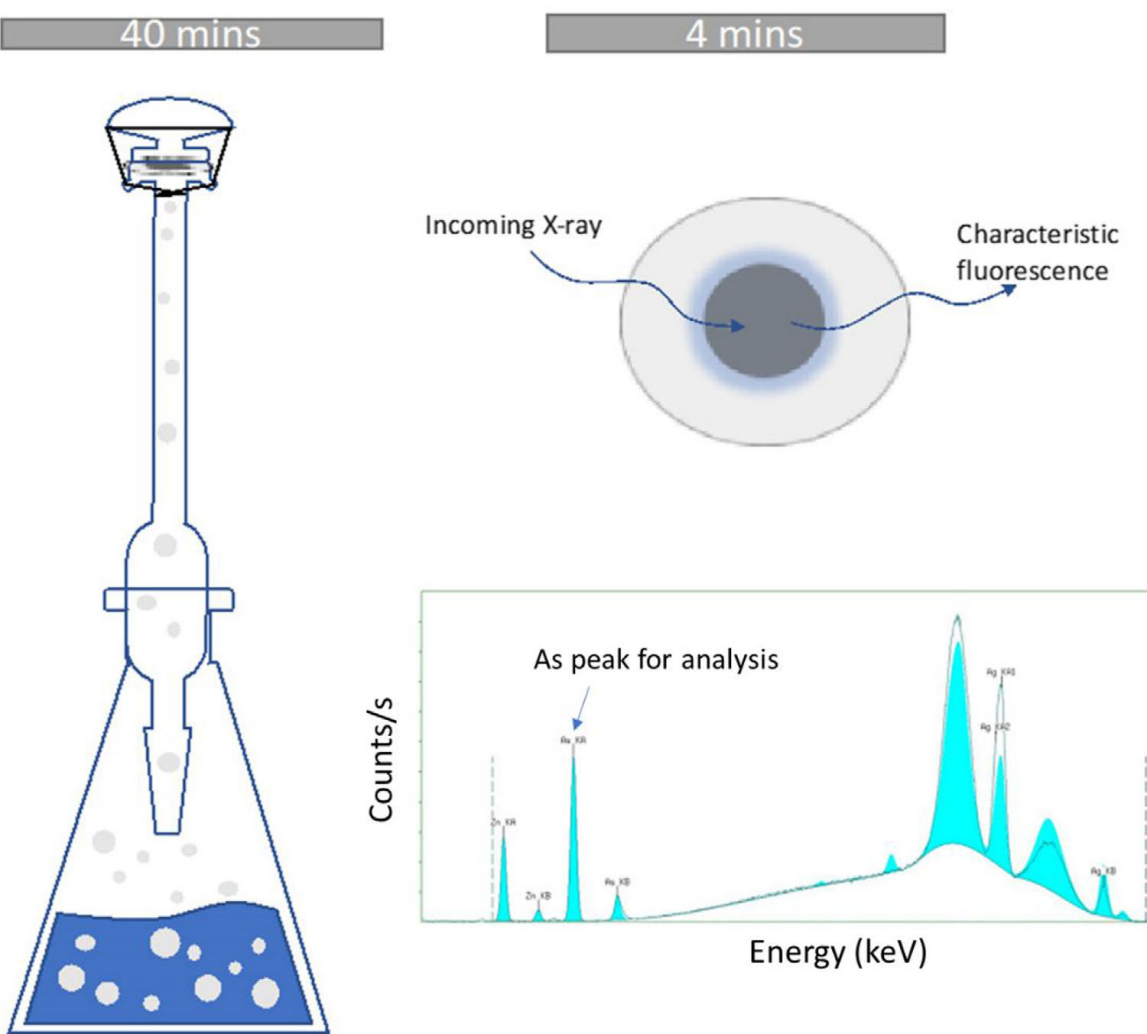
Schematic illustration of the approach for inorganic arsenic detection using X‐ray fluorescence spectroscopy. Inorganic arsenic is reduced to arsine gas and then collected on a filter membrane. Then the filter membrane is scanned by an X‐ray fluorescence spectrometer

Fifteen milliliters of liquid sample is added into a 150 mL Erlenmeyer flask. 0.25 mL of SnCl_2_ (40%, m/V) and 10 mL of H_2_SO_4_ (1.5 M) were added to the flask. Three grams of zinc powder was added to generate arsine gas. Mercury bromide test strip and silver nitrate test strips were cut into discs and installed onto the holder of the outlet tube to capture rising arsine gas. For silver nitrate test strips, 10 µL of silver nitrate solution was used to wet the filter paper and placed onto the gas outlet tube for arsine capture. After 30 minutes, discs are removed and analyzed by XRF. All steps are presented in Figure 1.

### 2.3 Comparison of standard curves between silver nitrate and mercury bromide

Different volumes (0, 0.05, 0.1, 0.15, 0.25, 0.5) of arsenic stock solutions (1 µg/mL) are added with DI water to achieve a final volume of 15 mL. Ten milliliters of H_2_SO_4_, 0.25 mL of SnCl_2_ were spiked into the solution. 1 g of zinc powder was added to initiate arsine gas formation. After 45 min, the disc with arsine gas is analyzed by XRF. The procedure is the same for both silver nitrate and mercury bromide test discs. The LOD and LOQ were calculated based on the equations:

$$\mathrm{LOD}\;=\;\frac{3.3\times s}{b}$$

$$\mathrm{LOQ}\;=\;\frac{10\times s}{b}$$

where *s* is the standard deviation of the second‐lowest concentration and *b* is the slope of the calibration.

### 2.4 Determination of recovery of iAs from Apple juice matrix

Different volumes (0, 0.5, 0.1, 0.15, 0.25, 0.5) of arsenic stock solutions (1 µg/mL) are added to apple juice to achieve a final volume of 15 mL. 10 mL of 1 M H_2_SO_4_, 0.25 mL of SnCl_2_ were spiked into the solution. One gram of zinc powder was added to initiate arsine gas formation. After 45 min, the silver nitrate disc with arsine gas is analyzed by XRF. The apple juice inorganic arsenic concentration was validated by the previous paper as the same bottle of apple juice was used.^19^

### 2.5 Instrumentation

Arsenic analysis is performed using Malvern PAnalytical (Westborough, MA) Epsilon 4 X‐ray fluorescence spectrometer. The instrument operated at a voltage of 50 kV and 0.2 mA under air atmosphere using Ag X‐ray tube. The measurements of Arsenic Kα intensity were collected for 120 s. Sample spinner was used at two revolutions per second.

### 2.6 Statistical analysis

The mean and standard error were calculated for four replicate analyses. Means were compared by a two‐tailed Student's *t*‐test (two groups) or one‐way ANOVA followed by the Tukey multiple comparison test (more than two groups). The significant difference between the curves were calculated by paired t‐test. For all the tests, the statistical significance level was set at *p* < 0.05.

## 3 RESULTS AND DISCUSSION

### 3.1 Development and optimization of the Gutzeit method for inorganic arsenic

Tin chloride is used to reduce As(V) to As(III). As(III) formation into arsine gas is faster than As(V). Silver nitrate reacts with arsine gas and forms a dark grey color depending on the arsenic concentration.

$$H_{3}\mathrm{As}O_{4}+Sn^{2+}+2H+\to H_{3}\mathrm{As}O_{3}+Sn^{2+}+H_{2}O$$

$$\mathrm{As}{O_{3}}^{3-}+3\mathrm{Zn}+9H+\;\to \mathrm{As}H_{3}↑+3Zn^{2+}+3H_{2}O$$

$$\mathrm{As}H_{3}+3\mathrm{AgN}O_{3}\to \mathrm{AsA}g_{3}+3\mathrm{HN}O_{3}$$

Silver nitrate is a light‐sensitive compound, prefabricated test strips will degrade during storage.^22^ Test strips freshly prepared right before test would ensure its stability. The easiest way to prepare the fresh strips is to direct wet the strip with silver nitrate solution. We demonstrated here a wet strip was effective to capture the arsine gas.

### 3.2 Mercury bromide and silver nitrate comparison

**FIGURE 2:**
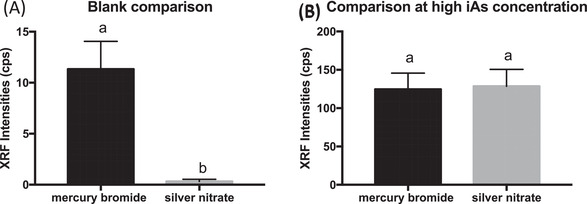
(A) X‐ray fluorescence peak intensity of silver nitrate and mercury bromide blanks. (B) X‐ray fluorescence peak intensity of silver nitrate and mercury bromide with 33 µg/L. Error bars are standard deviations (n = 3). Different letters for each column indicated a significant difference (*p* ≤ 0.05 one‐way ANOVA)

We compared the XRF intensities of silver nitrate and mercury bromide blank (Figure 2A) and arsenic concentration at 33 µg/L (Figure 2B). The blank of silver nitrate had a lower XRF intensity reading compared to the mercury bromide blank. XRF Spectrum analysis is important when there are high concentrations of multiple elements. The overlapping fluorescent lines could lead to the misidentification of peaks. Arsenic Kα line was chosen for the identification because the peak is significant and easily distinguished from other peaks. Another reason is arsenic Kβ line overlapped with other elements shown in Figures S1 and S2. The reason why silver nitrate has a lower blank reading is most likely due to cleaner spectra, less elements were present to alter deconvolution (Figures S3 and S4).

When arsenic concentration is at 33 µg/L, the two XRF intensities are not statistically significant. Though silver nitrate test disc intensity for iAs is significantly lower, this does not mean there is a lower amount of iAs. Quantification is dependent on the standard curve. This demonstrates silver nitrate is just as effective as mercury bromide at capturing arsine gas.

### 3.3 Reaction optimization

**FIGURE 3:**
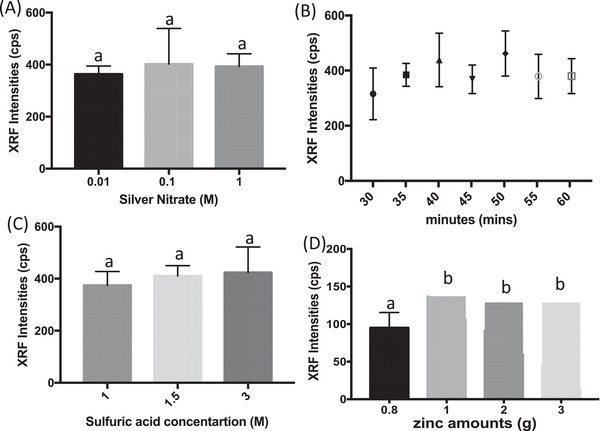
(A) X‐ray fluorescence peak intensities for 133 µg/L of arsenic collected with different silver nitrate concentrations. (B) X‐ray fluorescence peak intensities for arsenic collected at different min reaction times. (C) X‐ray fluorescence peak intensities for 133 µg/L of arsenic collected with different concentrations of sulfuric acid. (D) X‐ray fluorescence peak intensities for 133 µg/L of arsenic collected with different amounts of zinc powder. Error bars are standard deviations (n = 3). Different letters for each column indicated a significant difference (*p* ≤ 0.05 one‐way ANOVA)

The first optimization was done on silver nitrate test disc. The concentration of silver nitrate should be enough to efficiently capture arsine gas in sample but not excessive for disposal purposes. In Figure 3A, 0.01 M silver nitrate showed no significant difference compared to 1 M of silver nitrate. Silver nitrate (0.01 M) will not be saturated with 133 µg/L iAs and it is important to avoid having the test disc to be the limiting factor. Therefore, future experiments will utilize 0.01 M silver nitrate. Arsenic concentration of 133 µg/L was used for experiment optimization to ensure there are enough reagents to react with the iAs in unknown samples.

Reaction time was then optimized. It was observed the 35 min of reaction time was not statistically significant from 60 minutes (Figure 3B). Therefore, the following experiments will be performed at 35 minutes.

Sulfuric acid was used to generate redox reaction. Sulfuric acid (3 M) was used in our previous work. For green chemistry purposes, we decreased the acid concentration. It is observed 1 M was just as effective as 3 M in our study (Figure 3C). Referring to Figure 3D, 0.8 g of zinc powder has high variation and XRF intensity is significantly lower than 1, 2, and 3 g of zinc powder. This indicated the reaction was ongoing and 0.8 g of zinc was insufficient for reaction completion. 1 g of zinc powder is not statistically significant from 3 g of zinc powder. We deemed 1 g of zinc is sufficient to react with the concentration of acid used.

### 3.4 Standard curves for mercury bromide and silver nitrate

**FIGURE 4:**
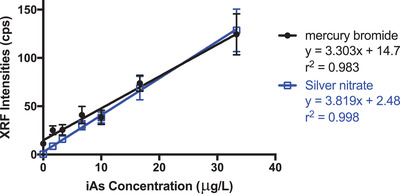
Plots of X‐ray fluorescence peak intensities as a function of inorganic arsenic concentration (0‐33.33 µg/L) for mercury bromide and silver nitrate test discs, respectively

The two standard curves were established from 0 to 33 µg/L. The LOD and LOQ for mercury bromide is 4.86 µg/L and 16.2 µg/L, respectively. As for silver nitrate, the LOD and LOQ are 1.02 and 3.40 µg/L, respectively. Silver nitrate has LOD and LOQ below 10 µg/L (the maximum allowable concentration in water set by FDA and WHO), which is better than mercury bromide because its LOQ exceeded the limit. The *t*‐test between the two slopes were calculated and *p* value is 0.0327, indicating the two reagents used for iAs capture is different. In Figure 4, there is an evident difference at low concentrations until there is an overlap at 16.67 µg/L. Less deviation is observed in silver nitrate, which is the reason for lower LOD and LOQ. To be noted, in our previous study,^19^ we reported the LOD and LOQ for mercury bromide was 1.9 and 5.7 µg/L, respectively. There are two reasons for the discrepancy. Firstly, we optimized the chemical reactions for silver nitrate, so the parameters were different from the previous study optimized for mercury bromide. To be fairly compared, we tested the mercury bromide under the same conditions as the silver nitrate. Secondly, two different XRF instruments were used (Epsilon 1 vs Epsilon 4). The Epsilon 4 is a high throughput that can handle multiple samples automatically versus Epsilon 1 which can only measure one sample at a time.

### 3.5 Apple juice spike and recovery analysis

**FIGURE 5:**
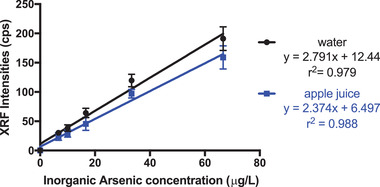
Plots of X‐ray fluorescence peak intensities as a function of iAs concentration (0‐66.66 µg/L) for water and apple juice using silver nitrate test discs, respectively (*p* < 0.08)

Using the optimized reaction conditions and a switch to silver nitrate test discs, standard curves for water and apple juice were established (Figure 5). At concentrations above 16.67 µg/L, the XRF intensity for apple juice is lower than the intensity of water. However, the two curves were not significantly different (*p* = 0.0862), indicating the apple juice matrix had some but not statistically significant influence. Table 1 shows the iAs recovery and relative standard deviation (RSD) from 0‐133 µg/L spiked in apple juice. The recovery was high (98% and above) for concentrations lower than 16.67 µg/L. Accurate quantification at around 10 µg/L is crucial because it is the set limit of iAs in beverages.^23^ The RSD represents reproducibility, which varied from 7.4‐24.5%. Though RSDs were relatively high for 10 and 16.67 µg/L, the average RSD (16.85%) is reasonable.

**TABLE 1:** Recoveries and relative standard deviation of spike additions of iAs to apple juice

Concentration added (µg/L)	Concentration found (µg/L)	Recovery (%)	RSD (%)
0.0	2.92	–	–
6.7	6.6 ± 1.1	98	17.5
10	10.2 ± 0.4	98	21.7
16.7	17.1 ± 2.4	98	24.5
33.3	39.2 ± 0.72	85	7.4
66.6	61.5 ± 8.4	92	16.8
133.3	134.4 ± 15.6	99	13.2

Mean concentrations reported as µg/L ± SD (n = 3).

In the unspiked apple juice, an iAs concentration was found to be at 2.25 µg/L using the method proposed in this study. This value is very close to the iAs concentration of 2.7 µg/L from previous work which analyzed the same brand of product.^19^

## 4 CONCLUSION

We concluded in this study silver nitrate has a great potential to be used in place of mercury bromide in iAs detection using XRF. It has the advantages of less toxicity and lower LOD and LOQ because of the lower XRF background interference. The standard curve for apple juice had a good recovery rate ranging from 85% to 99%, suggesting minimal matrix interferences. Prior study^19^ using mercury bromide developed a simple method using acetone to remove organic arsenic captured on the test paper. In the future study, we will further evaluate the organic arsenic interference, develop methods using greener organic acids with strong oxidizing abilities such as oxalic acid to replace sulfuric acid, as well as test more complex food matrices such as rice and seaweed.

## AUTHOR CONTRIBUTIONS

H.L. was associated with data curation, formal analysis (equal), investigation, and wrote the original draft preparation. H.D. was associated with formal analysis (equal). L.H. reviewed and edited the final manuscript.

## CONFLICT OF INTEREST

The authors declare there is no conflict of interest.

## Supporting information

Supporting information

## Data Availability

The data of this study is available upon reasonable request.

## References

[1] Ditzel EJ , Nguyen T , Parker P , Camenisch TD . Effects of arsenite exposure during fetal development on energy metabolism and susceptibility to diet‐induced fatty liver disease in male mice. Environ. Health Perspect. 2016;124(2):201–209. 10.1289/ehp.1409501 26151952 PMC4749082

[2] Von Ehrenstein OS , Poddar S , Yuan Y , et al. Children's intellectual function in relation to arsenic exposure. Epidemiology. 2007;18(1):44–51. 10.1097/01.ede.0000248900.65613.a9 17149142

[3] Ratnaike RN . Acute and chronic arsenic toxicity. Postgrad Med J. 2003;79(933):391–396. 10.1136/pmj.79.933.391 12897217 PMC1742758

[4] Bustaffa E , Stoccoro A , Bianchi F , Migliore L . Genotoxic and epigenetic mechanisms in arsenic carcinogenicity. Arch Toxicol. 2014;88(5):1043–1067. 10.1007/s00204-014-1233-7 24691704

[5] Studinski RCN , McNeill FE , Chettle DR , O'Meara JM . Estimation of a method detection limit for an in vivo XRF arsenic detection system. Phys Med Biol. 2005;50(3):521–530. 10.1088/0031-9155/50/3/009 15773727

[6] Ayotte JD , Medalie L , Qi SL , Backer LC , Nolan BT . Estimating the high‐arsenic domestic‐well population in the conterminous United States. Environ Sci Technol. 2017;51(21):12443–12454. 10.1021/acs.est.7b02881 29043784 PMC8842838

[7] Nachman KE , Ginsberg GL , Miller MD , Murray CJ , Nigra AE , Pendergrast CB . Mitigating dietary arsenic exposure: current status in the United States and recommendations for an improved path forward. Sci Total Environ. 2017;581‐582:221–236. 10.1016/j.scitotenv.2016.12.112 PMC5303536 28065543

[8] Liu J , Zheng B , Aposhian HV , Zhou Y , Chen ML , Zhang A , Waalkes MP . Chronic arsenic poisoning from burning high‐arsenic‐containing coal in Guizhou, China. Environ. Health Perspect. 2002;110(2):119–122. 10.1289/ehp.02110119 PMC1240722 11836136

[9] Baghel A , Singh B , Pandey P , Sekhar K . A rapid field detection method for arsenic in drinking water. Anal Sci. 2007;23(2):135–137. 10.2116/analsci.23.135 17297222

[10] Shehab H , Desouza ED , O'Meara J , et al. Feasibility of measuring arsenic and selenium in human skin using in vivo X‐ray fluorescence (XRF) ‐ a comparison of methods. Physiol Meas. 2015;37(1):145–161. 10.1088/0967-3334/37/1/145 26683849

[11] US Environmental Protection Agency . Arsenic and Clarifications to Compliance and NewSource Monitoring Rule: A Quick Reference Guide. 2001. EPA 816‐F‐01‐004. https://nepis.epa.gov/Exe/ZyPDF.cgi?Dockey=300065YM.txt

[12] Bralatei E , Lacan S , Krupp EM , Feldmann J . Detection of inorganic arsenic in rice using a field test kit: a screening method. Anal Chem. 2015;87(22):11271–11276. 10.1021/acs.analchem.5b02386 26506262

[13] Pan F , Tyson JF , Uden PC . Simultaneous speciation of arsenic and selenium in human urine by high‐performance liquid chromatography inductively coupled plasma mass spectrometry. J Anal At Spectrom. 2007;22(8):931–937. 10.1039/b703713a

[14] Kearns J , Tyson J . Improving the accuracy and precision of an arsenic field test kit: increased reaction time and digital image analysis. Anal Methods. 2012;4(6):1693–1698. 10.1039/c2ay05655k

[15] Kearns JK , Edson CB . Expanding quantification of arsenic in water to 0 µg L^−1^ with a field test kit: substituting 0.4% m/v silver nitrate as the colorimetric reagent, employing digital image analysis. Water Air Soil Pollut. 2018;229(3):1–7. 10.1007/s11270-018-3717-1

[16] Parsons C , Margui Grabulosa E , Pili E , Floor GH , Roman‐Ross G , Charlet L . Quantification of trace arsenic in soils by field‐portable X‐ray fluorescence spectrometry: considerations for sample preparation and measurement conditions. J Hazard Mater. 2013;262:1213–1222. 10.1016/j.jhazmat.2012.07.001 22819961

[17] Melamed D . Monitoring arsenic in the environment: a review of science and technologies with the potential for field measurements. Anal Chim Acta. 2005;532(1):1–13. 10.1016/j.aca.2004.10.047

[18] McIver DJ , Vanleeuwen JA , Knafla AL , et al. Evaluation of a novel portable X‐ray fluorescence screening tool for detection of arsenic exposure. Physiol Meas. 2015;36(12):2443–2459. 10.1088/0967-3334/36/12/2443 26536141

[19] Zhang Z , Lin H , Ma C , et al. Integrating the Gutzeit method with X‐ray fluorescence spectroscopy for rapid quantification of inorganic arsenic in selected beverages. Food Control. 2021,121:107588. 10.1016/j.foodcont.2020.107588

[20] Arshad M , Sadef Y , Shakoor MB , et al. Quantitative estimation of the hydroquinone, mercury and total plate count in skin‐lightening creams. Sustain. 2021;13(16):1–13. 10.3390/su13168786

[21] Patel UN , Patel UD , Khadayata AV , et al. Assessment of neurotoxicity following single and co‐exposure of cadmium and mercury in adult zebrafish: behavior alterations, oxidative stress, gene expression, and histological impairment in brain. Water Air Soil Pollut. 2021;232(8). 10.1007/s11270-021-05274-1

[22] Kar S , Majumdar RK , Chaudhuri MK . Low cost quantitative arsenic determination kit: a method by using silver nitrate. J Geol Soc India. 2014;84(1):35–40. 10.1007/s12594-014-0108-0

[23] WHO/UN . Evaluations of the Joint FAO /WHO Expert Committee on Food Additives (JECFA). 2016, *1* (June), 5–6.

