# Supplementary material for: Toward a greener approach to detect inorganic arsenic using the Gutzeit method and X‐ray fluorescence spectroscopy
Source: Anal Sci Adv. 2022 Sep 16;3(9-10):262–8. doi: 10.1002/ansa.202200014 (PMC10989649; doi:10.1002/ansa.202200014)
Supplement: Supplementary file 1 — Supporting information [file ANSA-3-262-s001.pdf]

Supporting information for towards a greener approach to detect inorganic arsenic using the Gutzeit method and X-ray fluorescence spectroscopy

Helen Lin, Haochen Dai, Lili He

Department of Food Science, University of Massachusetts, Amherst, Massachusetts 01003, United States

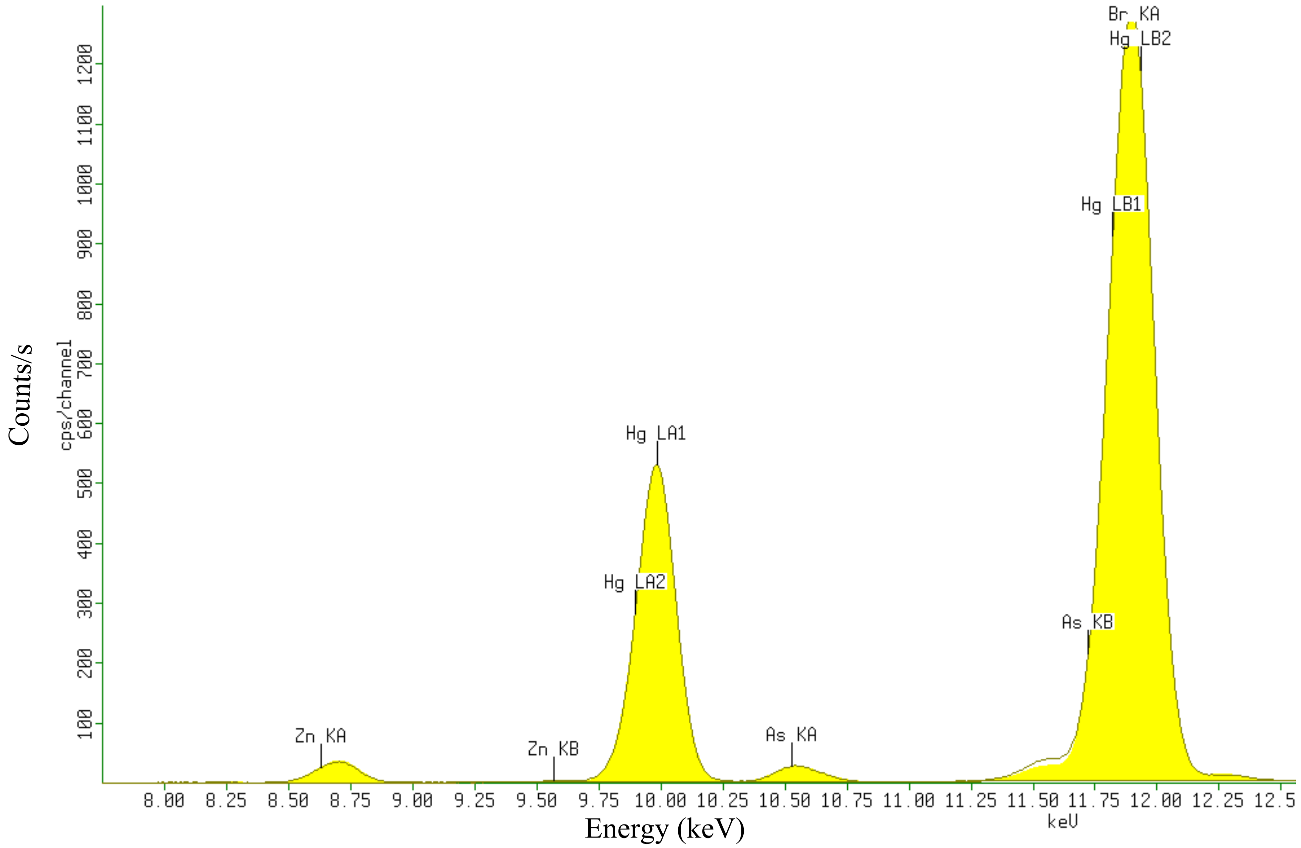

Figure S1. X-ray fluorescence spectrum of mercury bromide test strip with 133 μg/L inorganic arsenic captured.

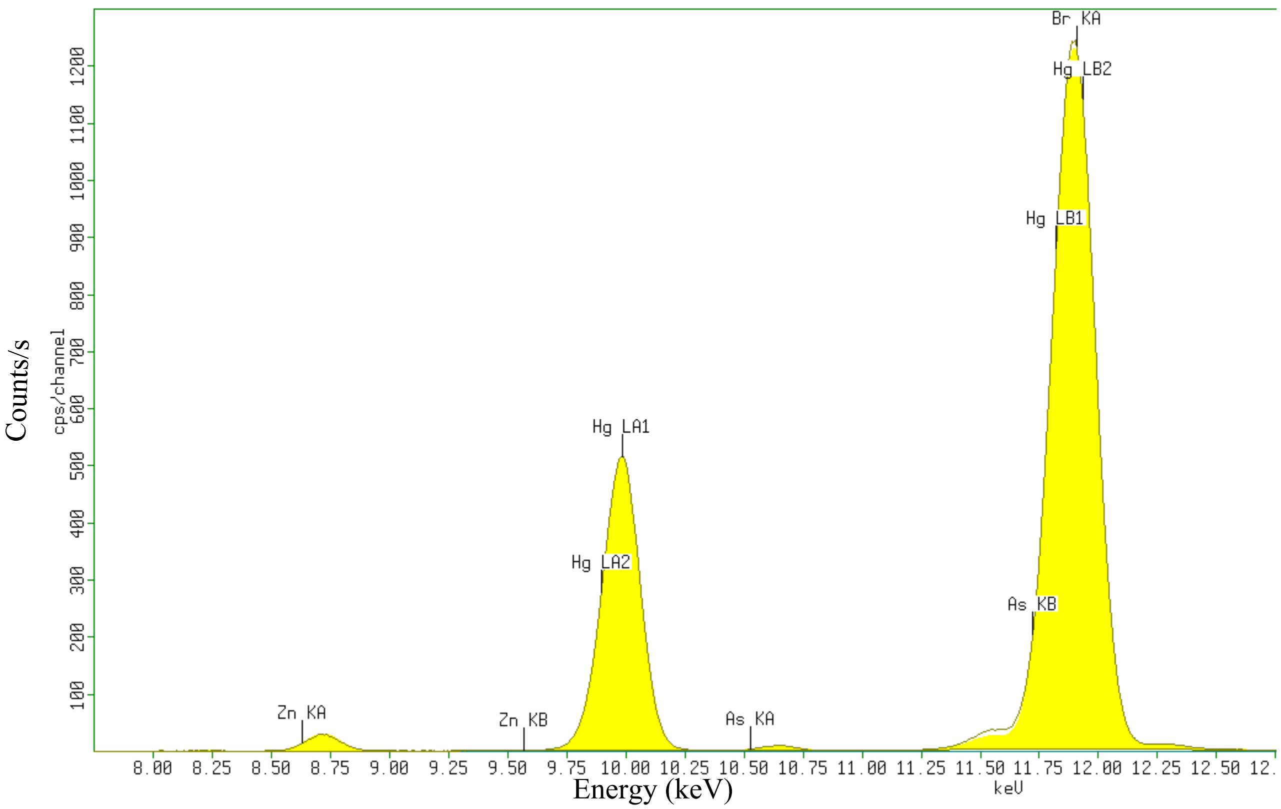

Figure S2. X-ray fluorescence spectrum of mercury bromide test strip with 0 μg/L inorganic arsenic captured.

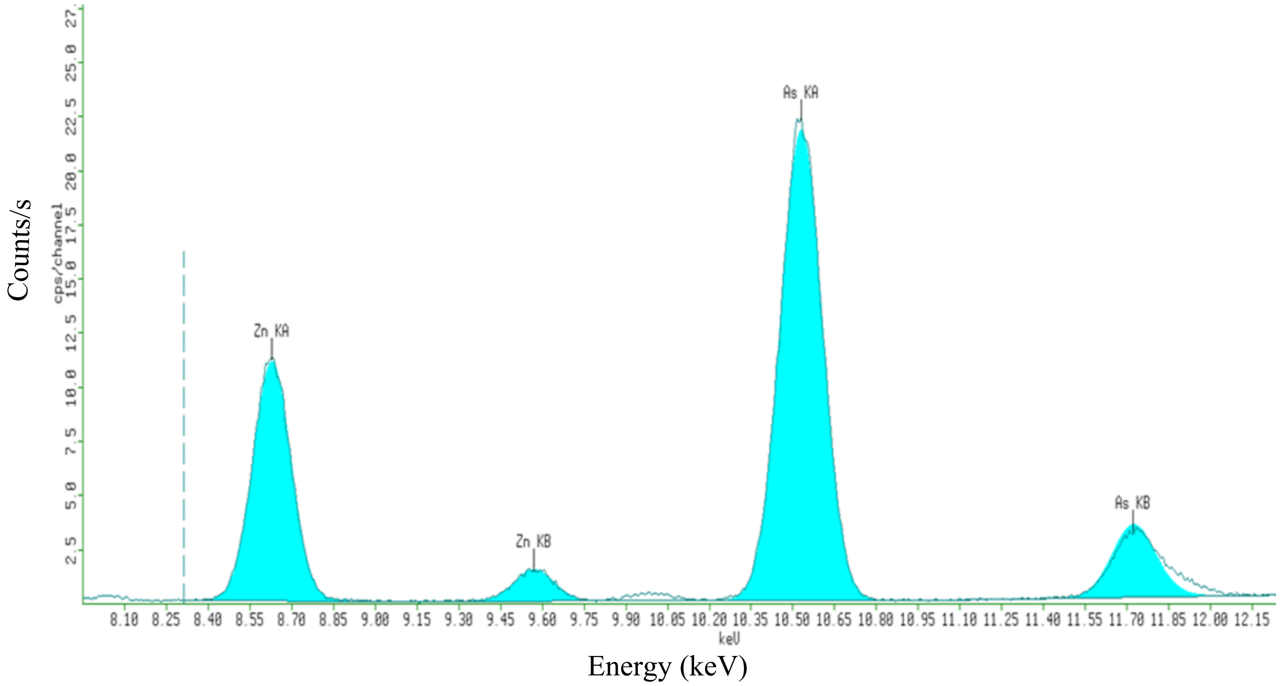

Figure S3. X-ray fluorescence spectrum of silver nitrate test strip with 133 μg/L inorganic arsenic captured.

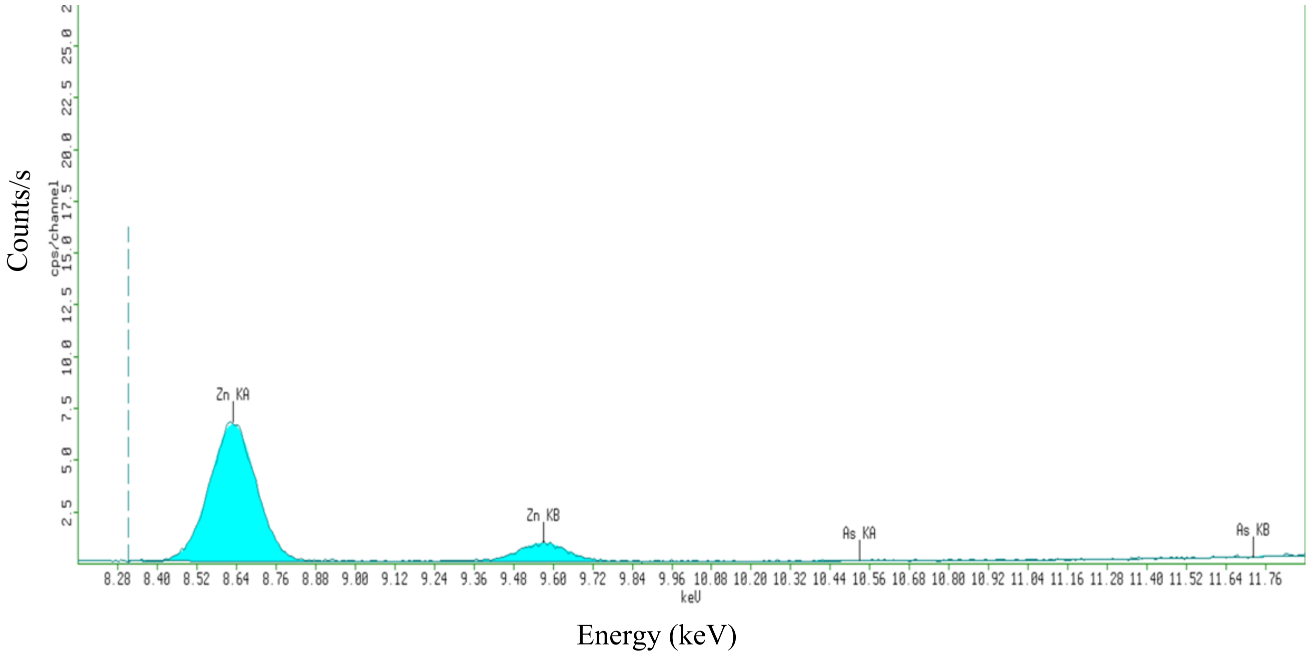

Figure S4. X-ray fluorescence spectrum of silver nitrate test strip with 0 μg/L inorganic arsenic captured.
